# Combined association of gait speed and processing speed on cardiometabolic disease mortality risk in the US older adults: a prospective cohort study from NHANES

**DOI:** 10.3389/fnagi.2025.1537413

**Published:** 2025-06-13

**Authors:** Hang Yang, Ye Zhou, Xiaoying Wang, Xiaoming Xu

**Affiliations:** ^1^Department of the Rehabilitation Medicine, The First Affiliated Hospital of Zhejiang Chinese Medical University, Hangzhou, Zhejiang Province, China; ^2^Department of Cardiology, The First Affiliated Hospital of Zhejiang Chinese Medical University, Hangzhou, Zhejiang Province, China

**Keywords:** gait speed, processing speed, combined association, cardiometabolic diseases, mortality

## Abstract

**Background:**

Gait speed and processing speed, as measured by the Digit Symbol Substitution Test (DSST), are important indicators of health in older adults, with their potential impact on mortality risk. However, their combined effects on cardiometabolic disease (CMD) mortality remain unclear.

**Objective:**

This study investigates how gait speed and cognitive function, individually and combined, influence CMD-specific and all-cause mortality in older adults.

**Methods:**

Data were obtained from the National Health and Nutrition Examination Survey 1999–2002, with mortality follow-up linked to the National Death Index. Gait speed was measured by the timed 20-foot walk and processing speed was assessed using the DSST. Then the combined Gait-DSST groups were created and the Cox proportional hazards regression (HR) models were applied to examine their associations on CMD-specific and all-cause mortality, as well as the subgroup analyses stratified by age, sex and education.

**Results:**

A total of 2,482 participants aged ≥60 years were included in the study with a median follow-up of 175 months, during which 587 CMD-specific deaths and 1,627 all-cause deaths were recorded. The slow gait was significantly associated with increased risk of CMD mortality, while low processing speed was only significantly associated with increased all-cause mortality risk. When analyzing the combined groups, individuals with slow gait and high processing speed exhibited a 86% increased risk of CMD mortality (HR = 1.86, 95% CI: 1.29, 2.68). However, the group with poor gait and processing speed had a twofold increased risk for all-cause mortality (HR = 2.01, 95% CI: 1.69, 2.39). The significant associations between slow gait with low processing speed and CMD mortality was more likely to be in age<75 years, male, and less-educated populations.

**Conclusion:**

Slow gait is a significant predictor of CMD-specific mortality in older adults, largely independent of processing speed. Routine screening of gait speed and DSST performance should be prioritized in clinical and public health settings. Future intervention studies should aim at elucidating the biological and behavioral mechanisms linking physical and cognitive function to CMD outcomes.

## 1 Introduction

Cardiometabolic diseases (CMD), including heart disease, stroke, and diabetes, are among the leading causes of death in older adults worldwide (World Health Organization, n.d.). Cardiometabolic health among the US adults saw a marked decline from 1999–2000 to 2017–2018, with only 6.8% achieving optimal cardiometabolic health by the latter period (O'Hearn et al., 2022), making CMD a pressing public health issue (O'Hearn et al., 2021).

The aging process is accompanied by atrophy in various brain regions, leading to declines in both mobility and cognitive abilities, which are indicators of frailty in older adults (Lauretani et al., 2017). CMD is bidirectionally associated with physical and cognitive domains (Zhang et al., 2023; Beauchet et al., 2018). The most widely used Cardiovascular Health Study (CHS) scale has been expended to include cognitive and emotional assessments, thereby redefining frailty (Afilalo, 2011). Physical and cognitive changes may share underlying biological mechanisms to reflect the aging and neurodegeneration (Cao et al., 2022). Systematic reviews and meta-analyses highlight the substantial potential of cognitive-motor dual-task training in improving disability and enhancing the quality of life in older adults (Wollesen et al., 2020). However, despite increased research attention, most studies have predominantly centered on all-cause mortality, with limited exploration of CMD-specific mortality (Chen C. et al., 2023).

Verghese et al. (2013) introduced the concept of Motoric Cognitive Risk (MCR) syndrome, defined by the coexistence of slow gait and subjective cognitive decline, positioning it as a pre-dementia syndrome, with many studies linking it to dementia, falls, and all-cause mortality (Maggio and Lauretani, 2019). However, systematic analyses of other combinations of gait speed and cognitive characteristics remain limited. Moreover, inconsistencies in defining gait and cognitive thresholds contribute to study heterogeneity (Verghese et al., 2013; Meiner et al., 2020).

While the association between slow gait and mortality is widely accepted, the relationship between cognitive decline and mortality risks remains controversial (An et al., 2018; Wang et al., 2024). Prior studies have used different cognitive domains and assessment tools such as Mini-Mental State Examination (MMSE), Montreal Cognitive Assessment (MoCA), or Digit Symbol Substitution Test (DSST) (An et al., 2018; Adjoian Mezzaca et al., 2022). Yet, only a few studies have preliminarily explored the interaction between gait and cognitive domains in predicting cardiovascular disease (CVD) risk factors or events, yielding mixed results (Sekhon et al., 2019; Iqbal et al., 2022; Beauchet et al., 2018). Variations across sex, age, and education levels remain insufficiently clarified, underscoring the need for further investigation.

To address these gaps, this study utilizes 20 years of follow-up data from the National Health and Nutrition Examination Survey (NHANES) to examine gait speed and cognitive domains, particularly in processing speed, as measured by the DSST, with the CMD-specific mortality in older adults. The DSST is a validated tool from the Wechsler Adult Intelligence Scale (WAIS-III) that primarily captures processing speed, sustained attention, and elements of working memory (Wechsler, 1997). Although not a global cognition, the DSST has been widely used in aging and epidemiologic research as a sensitive indicator of early cognitive change (Bienias et al., 2003). The goal of this study is to provide evidence for early CMD risk identification and targeted interventions in high-risk populations by these key functional domains.

## 2 Methods

### 2.1 Study population

The NHANES is a program designed to assess the health and nutritional status of the United States (US) since the early 1960s conducted by the National Center for Health Statistics (NCHS). Moreover, it plays a primary role in collecting extensive examinations for the older people (≥60 years) to increase the knowledge of the aging population through a stratified, multistage probability sampling. In this cohort study, data from the 1999–2002 cycles were used, as both gait speed and cognitive function measures were available in these cycles. The NCHS Ethics Review Board subsequently approved the NHANES protocols, and all participants provided written informed consent at enrollment. Through linkage with the National Death Index (NDI), we investigated the associations of gait speed and cognitive function with mortality. Participants were excluded if follow-up data, gait speed, and cognitive functioning test were unavailable.

### 2.2 Gait speed measurement

The measurement of gait speed was based on the timed 20-foot walk at the NHANES Mobile Examination Center (MEC). Participants were instructed to walk at a normal pace, but they were not allowed to hold onto another person for support. A 20-foot (6.15-meter) test track was marked, with tape indicating the start and end points. Timing began when one foot crossed the start line and contacted the floor, and it stopped when one foot crossed the finish line and made contact with the floor. Gait speed was calculated as: Gait speed (m/s) = 6.15 (m) / timed 20-foot walk (s). We defined slow gait as a walking speed below 0.8 m/s, based on previous studies suggesting this threshold is commonly used to identify frailty and sarcopenia (Mayhew et al., 2023; Bhasin et al., 2020).

### 2.3 Cognitive performance (processing speed)

The cognitive performance was assessed using the DSST, which primarily reflects processing speed, along with elements of sustained attention and working memory. A maximum score of 133 points can be gained when participants match numbers with the corresponding symbols within 120 s. However, a standardized rating system for processing speed has not yet been established. In this study, to ensure the statistical power in each group after stratification by gait and processing speed, low processing speed was defined as a DSST score below the median of 42, based on previous studies (Zipperer et al., 2022; Liao et al., 2019).

### 2.4 Mortality

The mortality data were obtained through the linkage with the NDI where the latest mortality follow-up data has been updated through December 31, 2019. Therefore, the follow-up period for each participant began at the date of their participation in the NHANES survey and continued until the date of death or the most recent follow-up record (December 31, 2019). We explored the CMD-specific mortality which was based on the Tenth Reversion of International Classification of Disease (ICD-10), including diseases of heart (I00-I09, I11, I13, I20-I51), cerebrovascular diseases (I60-I69) and diabetes mellitus (E10-E14) (Li et al., 2022; Zhang et al., 2023), as well as the mortality from all causes.

### 2.5 Covariates

The selection of possible potential confounders was based on clinical practice and previous studies. The sociodemographic variables included age, sex, education, and family poverty income ratios (PIR) collected through a Computer-Assisted Personal Interviewing system. Biological sex included male and female. Education level was classified as < 9 years, 9–12 years and ≥ 12 years. PIR was classified as low level (<1.3), medium level (1.3–3.5), and high level (≥3.5). Body mass index (BMI, kg/m^2^) was classified as obesity (≥30 kg/m^2^) and non-obesity (<30 kg/m^2^). Physical activity was classified as more, less and same activity based on “Compared activity with others same age.” Balance issues were based on the self-reported “Dizzy, falling and falling problems past year.” Smoking was classified as smokers (smoked at least 100 cigarettes in their life, whether smoking now or not) and non-smokers (smoked less than 100 cigarettes in their life). Alcohol use was classified as yes or no based on the answer to “Had at least 12 alcoholic drinks per year?”

The laboratory examinations were as follows. C-reactive protein (CRP, mg/dL) was quantified by latex-enhanced nephelometry method. Total cholesterol (TC, mg/dL) was measured enzymatically in serum or plasma with the absorbance of 500 nm, while a direct immunoassay technique was used for high-density-lipoprotein (HDL) cholesterol with the absorbance of 600 nm. Then the non-HDL cholesterol was calculated as TC minus HDL. Glycohemoglobin (%) was analyzed by a fully automated glycohemoglobin analyzer, which utilizes the principle of boronate affinity high performance liquid chromatography.

The BMI and the blood pressure (systolic and diastolic blood pressure, SBP and DBP) were measured in the MEC.

Medical condition provided the self-reported physician diagnosis of hypertension, diabetes, heart failure, coronary heart disease, angina pectoris, heart attack, stroke and cancer. Participants with heart diseases or stroke were considered to have a chronic history of CVD.

A comprehensive CMD-related risk factor (CMDRF) was further created (O'Hearn et al., 2022), including obesity (BMI ≥ 30 kg/m^2^), hyperlipidemia (non-HDL cholesterol level ≥160 mg/dL), hypertension (the SBP ≥ 140 mmHg or DBP ≥ 90 mmHg or self-reported physician diagnosis), and diabetes (glycohemoglobin>6.5% or self-reported physician diagnosis). One point is given for each abnormal indicator of CMDRF, with a maximum score of 4 points.

Some covariates were missing at random, including education level (missing 0.04%), PIR (missing 10.91%), physical activity (missing 0.04%), balance (missing 0.04%), smokers (missing 0.04%), alcohol use (missing 1.21%), CRP (missing 4.47%), self-reported hypertension (missing 0.12%), the measurement of systolic pressure and diastolic pressure (missing 1.37%), glycohemoglobin (missing 2.98%), BMI (missing 3.02%), TC (missing 5.08%), HDL-cholesterol (missing 5.08%). Missing values were imputed using the Multivariate Imputation by Chained Equations (MICE) algorithm, with Bayesian ridge regression as the estimator in the iterative chained equations (Van Buuren and Groothuis-Oudshoorn, 2011). Imputation models were selected based on variable types, including predictive mean matching for continuous data, logistic regression for binary variables, and polytomous or proportional odds models for categorical data. The imputation model included all analytic variables, assuming data were missing at random. The comparison between missing and complete data was shown in Supplementary Table 1.

### 2.6 Statistical analysis

To represent the civilian, non-institutionalized US population, the analytic guidelines of NHANES survey suggested the use of sampling parameters to avoid biased estimates. The four-year MEC weight should be used in this study. Firstly, the categorical variables were represented as unweighted number (percentage), and the continuous variables are presented as mean (standard error, SE). ANOVA, Kruskal–Wallis, and chi-squared tests were applied to compare differences across groups.

A multivariable Cox proportional hazards regression analysis was utilized to examine the relationship between the separate variables and mortality. Furthermore, a four-level combined Gait-DSST variable was created as: Group 1, normal gait and high DSST; Group 2, normal gait and low DSST; Group 3, slow gait and high DSST; Group 4, slow gait and low DSST. The Cox model was further used to investigate the combined effects of Gait-DSST performance and mortality, as well as an interaction analysis for the gait and processing speed. The selection of confounding variables for three models was based on the clinical relevance and previous studies (Wang et al., 2024; Li et al., 2022). Model 1 was adjusted for sociodemographic covariates (age, sex, education, PIR); Model 2 was additionally adjusted for health lifestyles and medical conditions (physical activity, balance, smokers, alcohol use, CRP and history of cancer); Model 3 was further included adjustments for history of CVD and CMDRF.

To account for competing risks, we used the Fine-Gray subdistribution hazard model to estimate cause-specific cumulative incidence and subdistribution hazard ratios (sHR) for CMD mortality. Cumulative incidence curves (CICs) were constructed to visualize the cumulative incidence function for CMD-specific mortality.

Additionally, the subgroup analysis of the combined effects was carried out by age (<75 vs. ≥ 75 years) (Mullin et al., 2022), because the prevalence of slow gait and cognitive decline increases significantly in individuals aged ≥75 years, sex (male vs. female), education level (≤12 years vs. >12 years), and likelihood ratio test was used for interaction.

Finally, we performed several sensitivity analyses to assess the robustness of the study findings. First, we treated DSST as a continuous variable to examine the association between each one-standard-deviation decrease in DSST and CMD mortality risk. Then, we used the lowest quartile (DSST score ≤30) as an alternative cutoff to classify processing speed and redefined the four Gait-DSST groups accordingly, and introduced another model (age and sex). Secondly, we excluded participants who died within the first 2 years of follow-up to minimize potential reverse causation. Further, we excluded all cases with missing data on covariates. Finally, we considered the another two important confounders (aspirin use and daily energy take).

All statistical analyses were performed using R Statistical Software (Version 4.2.2, http://www.R-project.org, The R Foundation) and Free Statistics analysis platform (Version 1.9.2, Beijing, China, http://www.clinicalscientists.cn/freestatistics). FreeStatistics is a software package provides intuitive interfaces for most common analyses and data visualization. It uses R as the underlying statistical engine, and the graphical user interface is written in Python. Most analyses can be done with just a few clicks. It is designed for reproducible analysis and interactive computing. Statistical significance was set at a two-sided *p* value < 0.05.

## 3 Results

### 3.1 Study population and baseline characteristics

**Figure 1:**
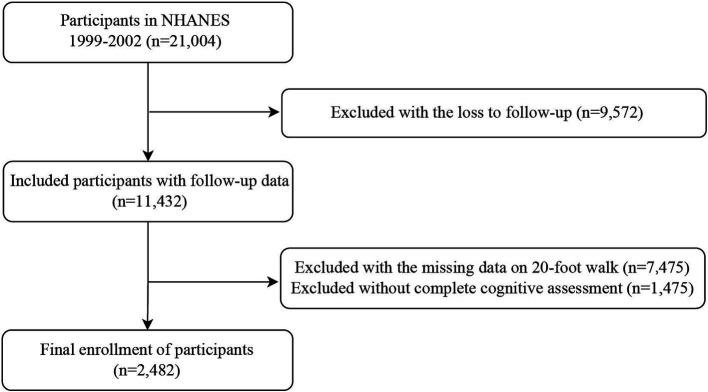
Flowchart of the screening and enrolment of study participants. NHANES, National Health and Nutrition Examination Survey.

A total number of 2,482 participants aged ≥ 60 years were included from a data pool of 21,004 participants, excluding the loss to follow-up (*n* = 9,572), unavailability of 20-foot walk time (*n* = 7,475) and incomplete cognitive function test (*n* = 1,475) (Figure 1).

The general characteristics of participants in the combined Gait-DSST groups are presented in Table 1, while we provided the weighted characteristics in Supplementary Table 2. The mean age of participants was 71.0 (0.2) years, with 1,225 (49.4%) being male. Participants with both poor gait and processing speed accounted for 20.9% and tended to be older [78.3 (0.3)], with lower education level, lower family income, less activity and more balance issues. They are more likely to have a higher level of CRP, more cases with history of CVD, higher CMDRF and higher all-cause mortality. Individuals with slower gait had the highest CMD mortality. The healthy group (normal gait and normal cognition) was younger [68.7 (0.2)], had high education and family income, more active, and more alcohol consumption. Additionally, they had fewer balance issues, lower CMDRF, lower history of CVD, and lower CRP levels, as well as the lowest all-cause and CMD mortality.

**Table 1:** Baseline characteristics of participants stratified by Gait-DSST in NHANES 1999–2002.

Variables	^a^Total (*n* = 2,482)	Slow gait (*n* = 710)		Normal gait (*n* = 1772)		
		Low DSST *n* = 519	High DSST *n* = 191	Low DSST *n* = 715	High DSST *n* = 1,057	*p*-value
Age (years)	71 (0.2)	75.3 (0.3)	73.1 (0.6)	70.8 (0.3)	68.7 (0.2)	< 0.001
Sex, male	1,225 (49.4)	237 (45.7)	59 (30.9)	422 (59)	507 (48)	< 0.001
Education level (years)						< 0.001
< 9	968 (39.0)	334 (64.4)	51 (26.7)	411 (57.5)	172 (16.3)	
9–12	612 (24.7)	93 (17.9)	58 (30.4)	157 (22)	304 (28.8)	
>12	902 (36.3)	92 (17.7)	82 (42.9)	147 (20.6)	581 (55)	
PIR						< 0.001
Low <1.3	674 (27.2)	246 (47.4)	48 (25.1)	256 (35.8)	124 (11.7)	
Median (1.3–3.5)	1,076 (43.4)	223 (43)	100 (52.4)	338 (47.3)	415 (39.3)	
High (≥3.5)	732 (29.5)	50 (9.6)	43 (22.5)	121 (16.9)	518 (49)	
Physical activity						< 0.001
More	1,272 (51.2)	193 (37.2)	88 (46.1)	368 (51.5)	623 (58.9)	
Less	268 (10.8)	98 (18.9)	47 (24.6)	59 (8.3)	64 (6.1)	
Same	942 (38.0)	228 (43.9)	56 (29.3)	288 (40.3)	370 (35)	
Balance disorder	704 (28.4)	238 (45.9)	74 (38.7)	190 (26.6)	202 (19.1)	< 0.001
Smokers	1,326 (53.4)	251 (48.4)	100 (52.4)	398 (55.7)	577 (54.6)	0.059
Alcohol use	1,494 (60.2)	264 (50.9)	101 (52.9)	414 (57.9)	715 (67.6)	< 0.001
CRP (mg/dL)	0.5 (0.02)	0.7 (0.05)	0.6 (0.06)	0.5 (0.04)	0.5 (0.02)	0.0012
History of cancer	465 (18.7)	104 (20)	38 (19.9)	106 (14.8)	217 (20.5)	0.017
History of CVD	555 (22.4)	170 (32.8)	47 (24.6)	174 (24.3)	164 (15.5)	< 0.001
Heart disease	480 (19.3)	144 (27.7)	41 (21.5)	145 (20.3)	150 (14.2)	< 0.001
Stroke	146 (5.9)	65 (12.5)	13 (6.8)	47 (6.6)	21 (2)	< 0.001
CMD risk factor (score)						< 0.001
0	274 (11)	39 (7.5)	12 (6.3)	76 (10.6)	147 (13.9)	
1	869 (35)	181 (34.9)	63 (33)	238 (33.3)	387 (36.6)	
2	849 (34.2)	166 (32)	63 (33)	263 (36.8)	357 (33.8)	
3	410 (16.5)	104 (20)	44 (23)	115 (16.1)	147 (13.9)	
4	80 (3.2)	29 (5.6)	9 (4.7)	23 (3.2)	19 (1.8)	
Obesity	768 (30.9)	161 (31)	82 (42.9)	203 (28.4)	322 (30.5)	0.002
Hyperlipidemia	1,131 (45.6)	228 (43.9)	81 (42.4)	334 (46.7)	488 (46.2)	0.602
Hypertension	1719 (69.3)	402 (77.5)	149 (78)	497 (69.5)	671 (63.5)	< 0.001
Diabetes	499 (20.1)	150 (28.9)	45 (23.6)	167 (23.4)	137 (13.0)	< 0.001
Gait speed (m/s)	0.9 (0.006)	0.6 (0.009)	0.7 (0.007)	1.0 (0.004)	1.1 (0.006)	< 0.001
DSST (score)	42.1 (0.4)	24.7 (0.5)	52.3 (0.7)	28.7 (0.4)	57.9 (0.4)	< 0.001
All-cause mortality	1,627 (65.6)	445 (85.7)	159 (83.2)	481 (67.3)	542 (51.3)	< 0.001
CMD mortality	587 (23.7)	191 (36.8)	71 (37.2)	161 (22.5)	164 (15.5)	< 0.001
Follow-up time, months	156.3 (1.4)	114.3 (3.2)	134.7 (5.0)	156.5 (2.7)	180.7 (1.9)	< 0.001

DSST, Digit Symbol Substitution Test, a cognitive test of processing speed; NHANES, National Health and Nutrition Examination Survey; PIR, poverty income ratio; CRP, C-reactive protein; CVD, cardiovascular and cerebrovascular disease; CMD, cardiometabolic disease.

a Data are presented as unweighted number (percentage) for categorical variables and mean (standard error) for continuous variables.

### 3.2 Separate associations with mortality

Over the 17-to 20-year follow-up period, 587 participants attributed to CMD-specific mortality, and 1,627 participants experienced all-cause mortality. The median follow-up time for all the participants was 175 months.

The associations between the separate variables (slow gait and low DSST) and CMD mortality differed (Table 2). Slow gait was associated with a 47% increased risk of CMD mortality (HR = 1.47, 95% CI: 1.21, 1.80). However, there was no association between low processing speed and CMD mortality after adjusting for sociodemographic covariates (age, sex, education, and PIR) (HR = 1.16, 95% CI: 0.95, 1.43) and remained non-significant in the fully adjusted model 3. In contrast, both slow gait and low processing speed were significantly associated with an increased risk of all-cause mortality in the fully adjusted model (Supplementary Table 3).

**Table 2:** The weighted association of separated gait speed, processing speed (DSST) and CMD mortality.

Models	Cardiometabolic disease mortality			
	^a^Death no. /total no.		HR (95%CI)	*p*-value
Gait speed	Normal gait	Slow gait		
Crude model	325/1772	262/710	2.4 (1.98, 2.90)	<0.001
Model 1	325/1772	262/710	1.73 (1.43, 2.08)	<0.001
Model 2	325/1772	262/710	1.56 (1.29, 1.89)	<0.001
Model 3	325/1772	262/710	1.47 (1.21, 1.80)	<0.001
Processing speed (DSST)	High DSST	Low DSST		
Crude model	235/1248	352/1234	1.81 (1.55, 2.10)	<0.001
Model 1	235/1248	352/1234	1.16 (0.95, 1.43)	0.138
Model 2	235/1248	352/1234	1.09 (0.89, 1.33)	0.865
Model 3	235/1248	352/1234	1.01 (0.82, 1.22)	0.983

a Death and total number of participants are presented as unweighted number.

DSST, Digit Symbol Substitution Test, a cognitive test of processing speed; CMD, cardiometabolic disease; HR, hazard ratio; CI, confidence interval. Model 1 was adjusted for age, sex, education, poverty income ratio (PIR); Model 2 was additionally adjusted for physical activity, balance, smokers, alcohol use, CRP and history of cancer; Model 3 was further adjusted for the history of cardiovascular and cerebrovascular diseases (CVD) and cardiometabolic disease risk factors (CMDRF).

### 3.3 Combined associations of gait-DSST groups on all-cause and CMD mortality

As shown in Table 3, after full adjustment in Model 3, individuals with slow gait yet high processing speed exhibited an 86% increased risk of CMD mortality (HR = 1.86, 95% CI: 1.29, 2.68), which was slightly higher than the risk for the group with poor gait and processing speed (HR = 1.40, 95% CI: 1.07, 1.83). This unexpected finding may reflect underlying subclinical pathology not yet manifested as cognitive decline. In contrast, participants with low processing speed yet normal gait did not exhibit a significant association with CMD mortality (HR = 1.09, 95% CI: 0.81, 1.47), suggesting that CMD mortality risk is largely independent of processing speed. No significant interaction was observed between gait and processing speed on CMD mortality.

**Table 3:** The weighted hazard ratios of CMD-specific and all-cause mortality by the combined Gait-DSST groups.

Variable	^a^Death no. /total no.	HR (95% CI)				P for interaction
		Crude	Model 1	Model 2	Model 3	
CMD Mortality	587/2482					
Gait-DSST						0.184
Group 1	164/1057	1 (Ref)	1 (Ref)	1 (Ref)	1 (Ref)	
Group 2	161/715	1.74 (1.33, 2.27)	1.27 (0.93, 1.73)	1.19 (0.87, 1.63)	1.09 (0.81, 1.47)	
Group 3	71/191	2.92 (2.08, 4.10)	2.33 (1.64, 3.31)	2.01(1.41, 2.86)	1.86 (1.29, 2.68)	
Group 4	191/519	2.88 (2.31, 3.58)	1.75 (1.38, 2.23)	1.58 (1.23, 2.03)	1.40 (1.07, 1.83)	
All-Cause Mortality	1627/2482					
Gait-DSST						0.032
Group 1	542/1057	1 (Ref)	1 (Ref)	1 (Ref)	1 (Ref)	
Group 2	481/715	2.06 (1.84, 2.31)	1.56 (1.39, 1.76)	1.56 (1.39, 1.76)	1.51 (1.35, 1.68)	
Group 3	159/191	2.70 (2.14, 3.40)	2.03 (1.61, 2.56)	1.81 (1.43, 2.29)	1.72 (1.35, 2.18)	
Group 4	445/519	3.54 (2.94, 4.26)	2.31 (1.97, 2.71)	2.12 (1.77, 2.53)	2.01 (1.69, 2.39)	

a Death and total number of participants are presented as unweighted number.

CMD, cardiometabolic disease; DSST, Digit Symbol Substitution Test, a cognitive test of processing speed; HR, hazard ratio; CI, confidence interval; Group 1: normal gait and high DSST; Group 2: normal gait and low DSST; Group 3: slow gait and high DSST; Group 4: slow gait and low DSST. Model 1 was adjusted for age, sex, education, poverty income ratio (PIR); Model 2 was additionally adjusted for physical activity, balance, smokers, alcohol use, C-reactive protein (CRP) and history of cancer; Model 3 was further adjusted for the history of cardiovascular and cerebrovascular diseases (CVD) and cardiometabolic disease risk factors (CMDRF).

Inconsistent with results of CMD mortality risk, all the combined groups showed increased all-cause mortality, with participants in the poor gait and processing speed group displaying the highest risk (HR = 2.01, 95% CI: 1.69, 2.39), more than double that of the healthy group. An interaction effect between gait and processing speed on all-cause mortality risk was identified (P for interaction = 0.032), underscoring the compounded impact of these factors.

**Figure 2:**
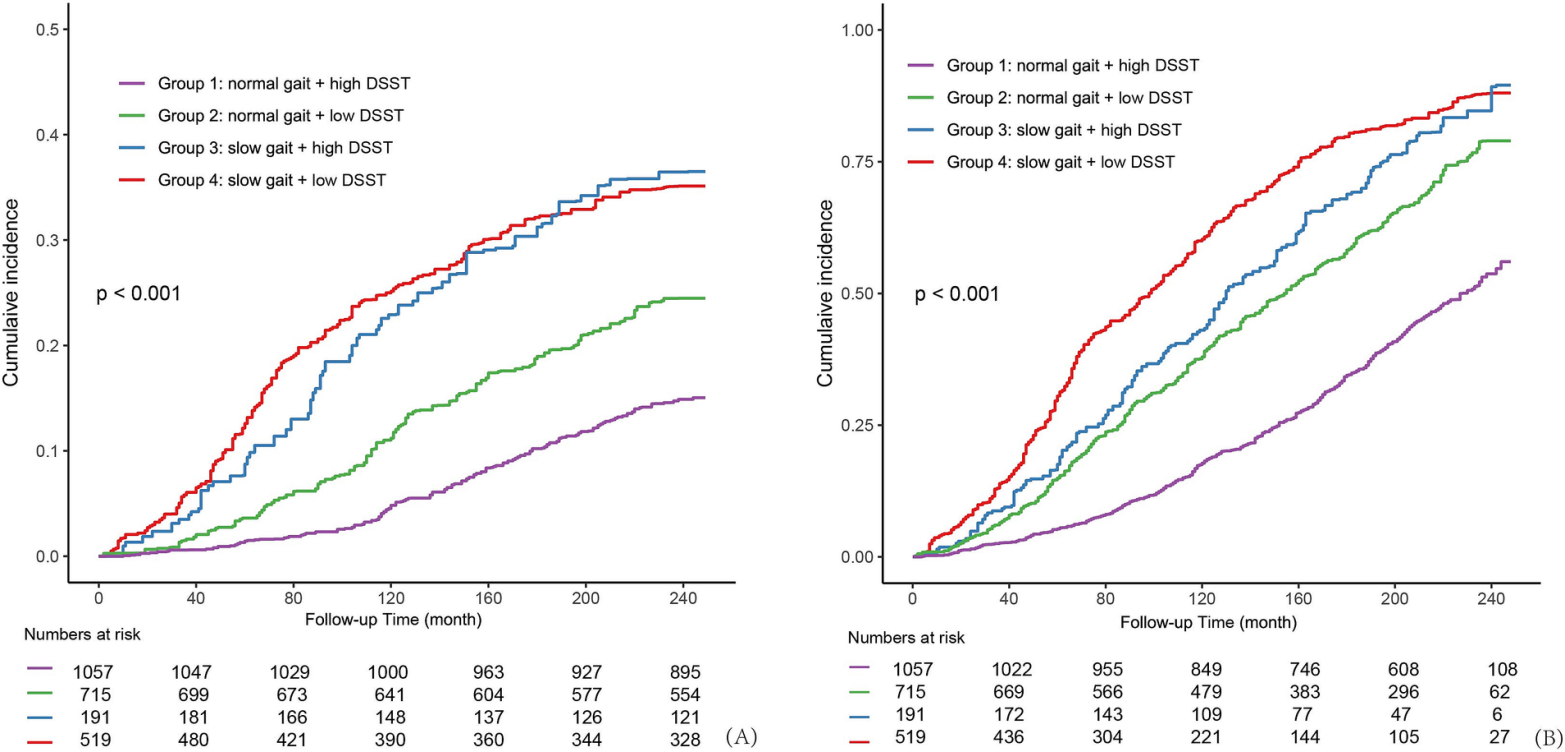
The weighted cumulative incidence curves among the four combined groups of gait and processing speed. The cumulative incidence curve shows the proportion of individuals experiencing CMD-specific and all-cause mortality rate (y-axis) over the follow-up period in months (x-axis) across four combined groups. Group 1: normal gait and high DSST; Group 2: normal gait and low DSST; Group 3: slow gait and high DSST; Group 4: slow gait and low DSST. DSST, Digit Symbol Substitution Test, a cognitive test of processing speed; CMD, cardiometabolic disease. **(A)** CMD mortality for Gait-DSST groups. Individuals with slow gait exhibited the higher cumulative CMD mortality risk with the slow gait and low DSST showing the highest risk. **(B)** All-cause mortality for Gait-DSST groups. Individuals with the both slow gait and low DSST exhibited the highest cumulative risk over time.

Figure 2 illustrates the weighted CICs for CMD mortality and all-cause mortality. These curves demonstrate that participants with slow gait faced consistently higher mortality risks compared to the normal gait groups. Participants with poor gait and processing speed exhibited the higher CMD mortality risks during short-term follow-up (<200 months), likely reflecting the synergistic effects of gait impairments and processing speed on early CMD risk. However, over longer follow-up periods (>200 months), the mortality risks for participants with slow gait, irrespective of processing speed, converged to similar levels. The results, presented in Supplementary Table 4, were consistent with those of the multivariable Cox proportional hazards model. In the fully adjusted Model 3, participants with slow gait—regardless of DSST performance—had significantly higher CMD mortality risk compared to the healthy group. Notably, the highest risk was observed in the slow gait yet high processing speed group (sHR = 1.91, 95% CI: 1.41, 2.59).

### 3.4 Subgroup analysis and sensitivity analysis

**Figure 3:**
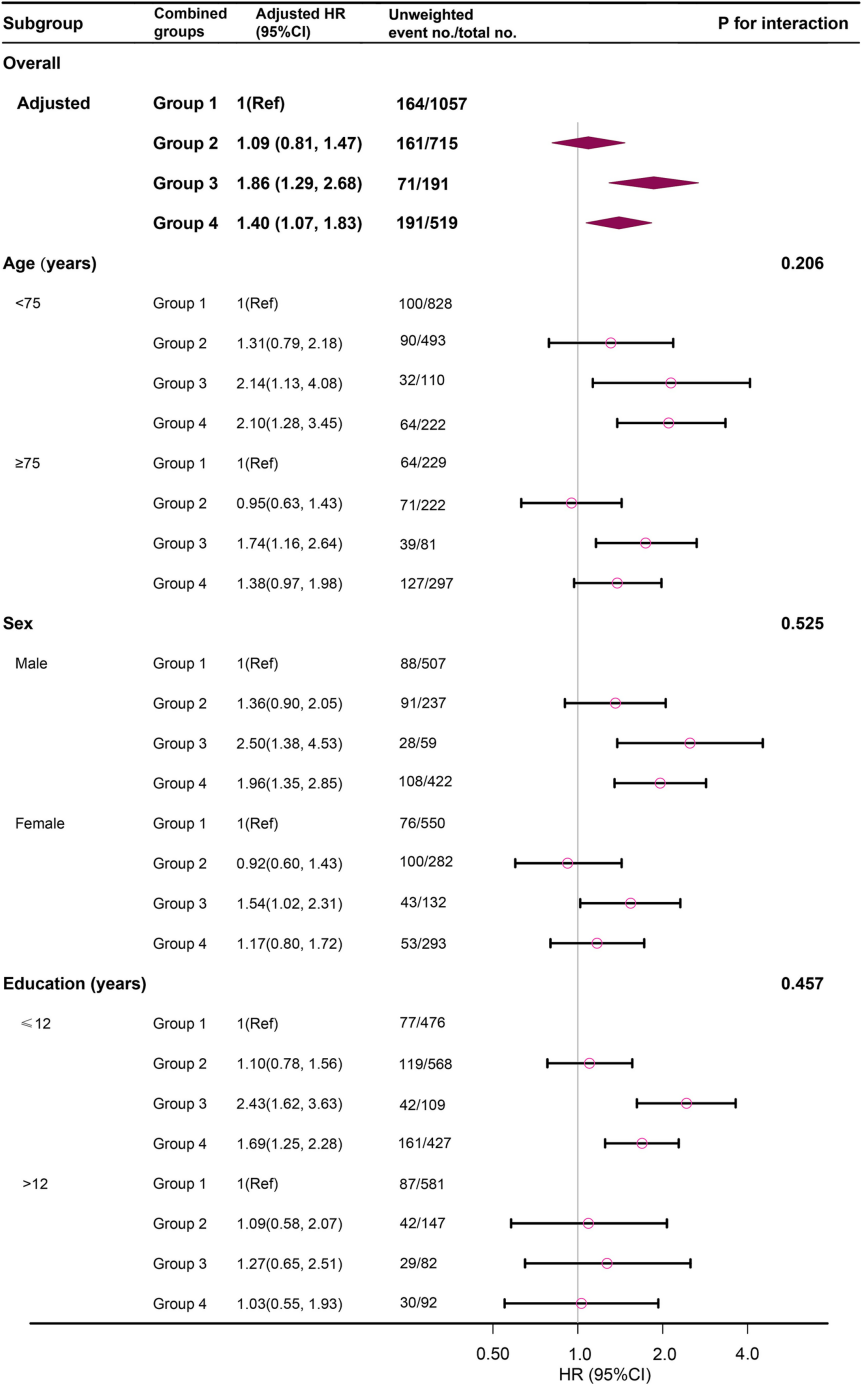
The weighted stratified analysis of the association between the combined Gait-DSST groups and CMD mortality risk. The stratification was adjusted for age, sex, education, family income, physical activity, balance, smokers, alcohol use, CRP, history of cancer, history of CVD, CMDRF except for the stratification factor itself. The circles represent the HRs and the horizontal lines represent 95% CIs. Diamonds represent the overall HR, and the outer points of the diamonds represent the 95% CI. Group 1: normal gait and high DSST; Group 2: normal gait and low DSST; Group 3: slow gait and high DSST; Group 4: slow gait and low DSST. DSST, Digit Symbol Substitution Test, a cognitive test of processing speed; CVD, cardiovascular and cerebrovascular diseases; CMD, cardiometabolic disease; CMDRF, cardiometabolic disease risk factors; HR, hazard ratio; CI, confidence interval.

Figure 3 illustrates the relationship between the combined Gait-DSST groups and CMD mortality across subgroups categorized by age (<75 years vs. ≥75 years), sex (male vs. female), and education level (≤12 years vs. >12 years). In all subgroups, the combined group with normal gait and low processing speed did not show a significant association with increased CMD mortality risk. However, among individuals <75 years, males, and those with lower education levels, the group characterized by slow gait and low processing speed was significantly associated with a higher risk of CMD mortality (even though interaction by age, sex, and education was not statistically significant). It is important to note that in populations with high education, this protective effect may need to be considered with caution due to the small number of CMD mortality.

On the other hand, the combined group effects were consistently and significantly associated with an increased risk of all-cause mortality across all subgroups, mirroring the overall observed trend (Supplementary Figure 1).

Sensitivity analyses support the primary results. Using a competing risk model, we found that the DSST score, whether treated as a continuous variable or redefined as low processing speed (DSST score≤30), was associated with CMD mortality in the crude model and the model adjusted for age and sex (Supplementary Table 4). However, no significant association was observed in the fully adjusted model, consistent with our previous findings. The other two were excluding participants who died within 2 years of follow-up (*n* = 2,389) and those with covariates missing (*n* = 2006) (Supplementary Tables 5–6). To address concerns regarding unmeasured confounding, we performed additional adjustments for aspirin use (yes/no) and daily energy intake (Kcal/day), with results remaining robust (Supplementary Table 7).

## 4 Discussion

This study explored the combined effect of gait speed and processing speed on CMD mortality. The results showed that individuals with slow gait had a significant higher CMD mortality risk compared to the healthy group. Even with high processing speed, slow gait was associated with an 86% increased risk (HR = 1.86, 95% CI: 1.29, 2.68). Interestingly, those with poor gait and processing speed showed a relatively lower CMD mortality risk (HR = 1.40, 95% CI: 1.07, 1.83). Low processing speed alone was not significantly associated with CMD mortality, suggesting that gait speed might be a more dominant driver of CMD mortality risk, while processing speed plays a secondary role. For all-cause mortality, all combined groups demonstrated higher risks compared to the healthy group, with the poor gait and processing speed group showing the highest risk (HR = 2.01, 95% CI: 1.69, 2.39).

The findings are largely consistent with previous research. Slow gait has been widely recognized as a predictor of higher mortality risks, frailty, hospitalization, dementia and disabilities (Dumurgier et al., 2017; Studenski et al., 2011; Kuo et al., 2006). A prospective cohort study from the three-city study in Dijon, France, reported that individuals aged ≥65 years with slow gait had nearly triple the risk of CVD mortality compared to those with normal gait (Dumurgier et al., 2009). Similarly, Curtis et al. found that slow gait was significantly associated with a higher risk of complications (e.g., stroke, infections) and increased in-hospital or 30-day mortality following cardiac surgery (Bergquist et al., 2018). Gait speed, recognized as the “sixth vital sign” (Merchant et al., 2021), has been identified as a stronger predictor of cardiovascular events than the New York Heart Association (NYHA) class (Tjam et al., 2012; Purser et al., 2006), reflecting systemic aging processes such as vascular stiffness, muscle weakness, and chronic inflammation—factors closely linked to CMD (Santos et al., 2017).

However, the association between cognitive performance and CMD-specific mortality remain uncertain. While cognitive decline, often assessed using the DSST, Animal Fluency (AnFl), and Consortium to Establish a Registry for Alzheimer’s Disease (CERAD), has been associated with all-cause mortality, its specific impact on CMD mortality has been inconsistent (Zhu and Liao, 2020; Adjoian Mezzaca et al., 2022). In this study, processing speed, defined using either the median or lowest quartile DSST score, was associated with CMD mortality in crude model and the model adjusted for age and sex. However, the association disappeared after further adjustment for education level and income. This aligns with the German ESTHER study (2000–present) (Perna et al., 2015), which found that lower cognitive scores (through Cognitive Telephone Screening Instrument) independently predicted all-cause mortality but were only significantly associated with CVD mortality only in age-and sex-adjusted models. Conversely, a 20-year follow-up longitudinal BLSA study using MMSE scores showed that cognitive decline remained significantly associated with CVD mortality even after adjusting for demographic, health behavior, and comorbidities (An et al., 2018). These discrepancies may be attributed to the low CMD events, variations in cognitive assessment tools, and differing definitions of cognitive decline. Additionally, demographic factors like education and income could mediate the relationship between cognitive function and CMD mortality risk.

This study provides new insights into the mechanisms underlying CMD mortality risk. It suggests that gait speed is a stronger and more consistent predictor of CMD mortality than processing speed, which appears to have a more indirect influence. This finding aligns with the EPIDOS cohort study in France, which reported that only slow gait and MCR were associated with mortality risk, while subjective cognitive impairment was not independently predictive (Beauchet et al., 2019). Although the study did not specifically analyze CVD or CMD mortality, it implies that mild cognitive impairment may be reversible (Petersen, 2011). In fact, gait slowing may precede cognitive decline (Wang et al., 2006), reflecting multisystemic dysfunctions that are closely related to CMD pathophysiology, including chronic inflammation and cardiovascular risk factors (Chen L. et al., 2023).

Additionally, DSST primarily assesses slowed cognitive processing speed, which might indirectly influence CMD through effects on decision-making, health management, or increased fall risks rather than serving as a direct driver (Sanders et al., 2017). O'Donnell et al. (2012) indicates that specific cognitive impairments involved in orientation, attention and calculation, recall, and copying designs, are among the strongest predictors of cardiovascular events. Over longer follow-ups, the impact of cognitive decline may be overshadowed by other mechanisms, limiting its ability to independently predict mortality risk. While DSST is not a comprehensive measure of global cognitive function, it captures a domain that is particularly sensitive to early cognitive decline and aging-related changes (Finkel et al., 2007). Processing speed is one of the earliest cognitive domains to deteriorate and is closely associated with both functional limitations and mortality in older adults (Kvale et al., 2010). Proust-Lima et al. (2007) found that while the MMSE and Benton Visual Retention Test (BVRT) were more sensitive to changes at lower levels of cognition, the DSST was more sensitive to detecting changes at higher levels.

An unexpected finding was that individuals with slow gait but high DSST performance had a higher CMD mortality risk than those with poor gait and processing speed. One possible explanation is competing risks—individuals with low processing speed may die from non-CMD causes (e.g., infections, falls, dementia-related complications) before CMD-related death occurs, leading to an underestimation of CMD mortality in this group when using cause-specific Cox models (Hageman et al., 2023). Alternatively, those with preserved processing speed may harbor early or subclinical cardiometabolic dysfunction, with gait slowing as the earliest manifestation, while still appearing cognitively intact. This aligns with the view of slow gait as an early marker of systemic aging and vascular burden (Tjam et al., 2012; Purser et al., 2006; Santos et al., 2017). Moreover, differential healthcare access or attention may play a role—cognitively intact individuals with physical frailty may be overlooked in CMD risk screening (Havranek et al., 2015). These findings underscore the need for targeted evaluation of cardiometabolic health even in older adults with isolated physical decline but preserved processing speed.

Regarding all-cause mortality, our findings align with most MCR-based studies of gait speed and cognition. Cohort studies in Europe, the United States, and Japan reported increased all-cause mortality risks of 50%, 87%, and 69%, respectively, in MCR groups (Maggio and Lauretani, 2019). Gait speed and cognitive performance interact to enhance the predictive power for all-cause mortality.

While no significant interactions were identified between combined groups and age, sex, or education in subgroup analysis, slight variations in CMD mortality risks were noted across populations. Slow gait with declined processing speed is more common in individuals over 75 years old. However, its relationship with CVDRF is not significant if aged≥75 years. In contrast, the CVD mortality risk is more prominent in individuals younger than 75 years (Sekhon et al., 2019). Female generally have a longer life expectancy than male (Ayers and Verghese, 2016). Bortone et al. (2022) found that male with slow gait and subjective cognitive impairment have a higher risk of mortality. A higher educational level is considered a protective factor, as it is associated with a lower prevalence of MCR (Verghese et al., 2014). Nevertheless, we must take into account the limitations posed by sample size and insufficient statistical power.

Although gait speed, processing speed, and CMD mortality are closely associated, the underlying biological mechanisms remain unclear and may involve shared pathological processes (Cao et al., 2022). Key contributors may include chronic inflammation, vascular and neurodegenerative changes, hormonal dysregulation, and metabolic disturbances (Panza et al., 2018). “Inflammaging” is closely linked to vascular aging and neurodegeneration, potentially leading to slower gait, cognitive decline, and elevated CMD risk (Franceschi et al., 2017). Inflammation may also trigger oxidative stress, hormonal axis dysregulation, and CVD (Robertson et al., 2013). Moreover, impaired neurovascular coupling may reduce cerebral perfusion, while neuroimaging studies have shown that white matter hyperintensities (WMHs) are predictive of both cognitive and physical decline and are associated with CVD pathology (Grueter and Schulz, 2012). The dysregulation of the hypothalamic–pituitary–adrenal (HPA), particularly elevated cortisol levels, may accelerate cardiovascular aging and impair central nervous function, contributing to both physical and cognitive decline (Lee et al., 2007). These interrelated mechanisms likely reflect systemic aging-related pathophysiology. Longitudinal and mechanistic studies are needed to validate these hypotheses and guide targeted intervention strategies.

This study has several limitations. First, as an observational study, causality between gait speed, cognitive function, their combined effects, and mortality cannot be established. Second, the research relies on NHANES public data. Baseline characteristics may have changed over time, and gait speed, while widely used to assess physical function in older adults, reflects only part of gait capacity, excluding more complex motor impairments. Similarly, the DSST was the only cognitive domain available in this cohort, limiting our ability to assess broader domains of cognition. However, DSST is more responsive to subtle changes in cognitive performance and is less influenced by education or cultural background, making it particularly suitable for population-based studies. Third, potential residual confounding due to unmeasured factors such as nutrition and medication use cannot be completely ruled out, although inclusion of caloric intake and aspirin use in sensitivity analyses did not materially alter the findings. Subgroup and sensitivity analyses using multivariable Cox and competing risk models further reduced potential bias. Finally, while secondary analyses from public data have limitations compared to original cohort studies, this study’s large sample size (2,482 participants) and long follow-up (17–20 years) enhance its strength. By applying NHANES sampling weights, we minimized bias and improved the generalizability of our findings on the combined effects of gait speed and cognition on mortality.

## 5 Conclusion

Slow gait is a significant predictor of CMD mortality in older adults, largely independent of processing speed. Routine screening of gait speed and DSST performance should be prioritized in clinical and public health settings. While our findings suggest potential benefits of health education and socio-economic support, these implications are inferential and should be interpreted with caution. Future intervention studies should aim at elucidating the biological and behavioral mechanisms linking physical and global cognition to CMD outcomes.

## Data Availability

The datasets presented in this study can be found in online repositories. The names of the repository/repositories and accession number(s) can be found in the article/Supplementary material.
