# Supplementary figures and images for: Combined association of gait speed and processing speed on cardiometabolic disease mortality risk in the US older adults: a prospective cohort study from NHANES
Source: Front Aging Neurosci. 2025 Jun 13;17:1537413. doi: 10.3389/fnagi.2025.1537413 (PMC12202421; doi:10.3389/fnagi.2025.1537413)

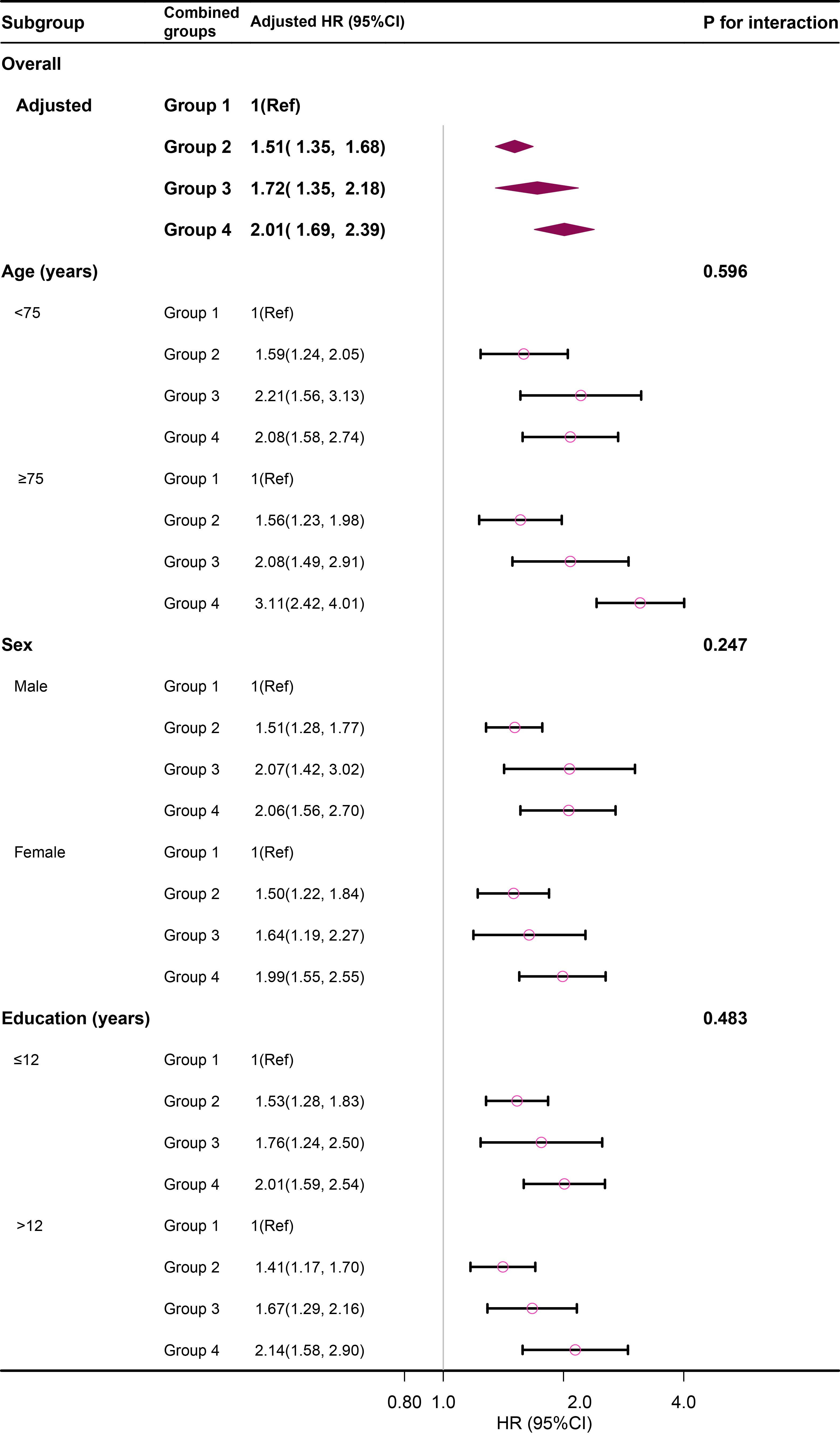

Supplement: Supplementary file 5 [file Image_1.jpeg]
