# Supplementary material for: Combined association of gait speed and processing speed on cardiometabolic disease mortality risk in the US older adults: a prospective cohort study from NHANES
Source: Front Aging Neurosci. 2025 Jun 13;17:1537413. doi: 10.3389/fnagi.2025.1537413 (PMC12202421; doi:10.3389/fnagi.2025.1537413)
Supplement: Supplementary file 6 [file Table_1.docx]

Supplementary Material

**Supplementary Table 1** Comparison of baseline characteristics between complete data and cases with missing in NHANES 1999-2002

| **Variables** | **^a^Total (n = 2482)** | **Case with missing data (n= 476)** | **Complete cases (n = 2006)** | **p-value** |
| --- | --- | --- | --- | --- |
| **Age, years** | 71 (0.2) | 71.7 (0.4) | 70.8 (0.2) | 0.031 |
| **Sex, male** | 1225 (49.4) | 224 (47.1) | 1001 (49.9) | 0.265 |
| **Education level, years** |  |  |  |  |
| < 9 | 968 (39.0) | 206 (43.3) | 762 (38) | 0.06 |
| 9-12 | 612 (24.7) | 117 (24.6) | 495 (24.7) |  |
| >12 | 902 (36.3) | 153 (32.1) | 749 (37.3) |  |
| **PIR** |  |  |  |  |
| Low <1.3 | 674 (27.2) | 154 (32.4) | 520 (25.9) | 0.004 |
| Median (1.3-3.5) | 1076 (43.4) | 206 (43.3) | 870 (43.4) |  |
| High (≥3.5) | 732 (29.5) | 116 (24.4) | 616 (30.7) |  |
| **Physical activity** |  |  |  |  |
| More | 1272 (51.2) | 234 (49.2) | 1038 (51.7) | 0.257 |
| Less | 268 (10.8) | 61 (12.8) | 207 (10.3) |  |
| Same | 942 (38.0) | 181 (38) | 761 (37.9) |  |
| **Balance disorder** | 704 (28.4) | 157 (33) | 547 (27.3) | 0.013 |
| **Smokers** | 1326 (53.4) | 256 (53.8) | 1070 (53.3) | 0.862 |
| **Alcohol use** | 1494 (60.2) | 262 (55) | 1232 (61.4) | 0.011 |
| **CRP, mg/dL** | 0.5 (0.02) | 0.6 (0.05) | 0.5 (0.02) | 0.411 |
| **History of cancer** | 465 (18.7) | 96 (20.2) | 369 (18.4) | 0.373 |
| **History of CVD** | 555 (22.4) | 111 (23.3) | 444 (22.1) | 0.577 |
| Heart disease | 480 (19.3) | 144 (27.7) | 41 (21.5) |  |
| Stroke | 146 ( 5.9) | 65 (12.5) | 13 (6.8) |  |
| **CMD risk factor, (score)** |  |  |  |  |
| 0 | 274 (11) | 50 (10.5) | 224 (11.2) | 0.746 |
| 1 | 869 (35) | 174 (36.6) | 695 (34.6) |  |
| 2 | 849 (34.2) | 153 (32.1) | 696 (34.7) |  |
| 3 | 410 (16.5) | 81 (17) | 329 (16.4) |  |
| 4 | 80 ( 3.2) | 18 ( 3.8) | 62 ( 3.1) |  |
| Obestiy | 768 (30.9) | 150 (31.5) | 618 (30.8) | 0.765 |
| Hyperlipdemia | 1131 (45.6) | 224 (47.1) | 907 (45.2) | 0.468 |
| Hypertension | 1719 (69.3) | 321 (67.4) | 1398 (69.7) | 0.338 |
| Diabetes | 499 (20.1) | 100 (21) | 399 (19.9) | 0.584 |
| **Gait speed, m/s** | 0.9 (0.006) | 0.9 (0.012) | 1 (0.006) | <0.001 |
| **DSST, score** | 42.1 (0.4) | 39.8 (0.9) | 42.7 (0.4) | 0.003 |
| **All-cause mortality** | 1627 (65.6) | 335 (70.4) | 1292 (64.4) | 0.014 |
| **CMD mortality** | 587 (23.7) | 114 (23.9) | 473 (23.6) | 0.864 |
| **Follow-up time, months** | 156.3 (1.4) | 145.6 (3.5) | 158.9 (1.6) | 0.004 |

**Abbreviations:** NHANES, National Health and Nutrition Examination Survey; PIR, poverty income ratio; CRP, C-reactive protein; CVD, cardiovascular and cerebrovascular disease; CMD, cardiometabolic disease; DSST, Digit Symbol Substitution Test, a cognitive test of processing speed;

^a^Data are presented as unweighted number (percentage) for categorical variables and mean (standard error) for continuous variables.

**Supplementary Table 2** The weighted baseline characteristics of participants stratified by Gait-DSST in NHANES 1999-2002

| **Variables** | **Total  (n = 36711585.7)** | **Slow gait speed** | | **Normal gait speed** | |  |
| --- | --- | --- | --- | --- | --- | --- |
|  |  | **Low DSST n= 6184445.9** | **High DSST n = 2980681.9** | **Low DSST n= 8021482.2** | **High DSST n = 19524975.6** | **p-value** |
| **Age (years)** | 70.5 (7.4) | 75.7 (7.5) | 72.9 (7.5) | 71.4 (7.0) | 68.2 (6.3) | <0.0001 |
| **Sex, male** | 16000680.8 (43.6) | 2185866.0 (35.3) | 714896.6 (24.0) | 4328238.6 (54.0) | 8771679.7 (44.9) | <0.0001 |
| **Education (years)** |  |  |  |  |  |  |
| < 9 | 10630466.9 (29.0) | 3646745.4 (59.0) | 709834.9 (23.8) | 3598995.9 (44.9) | 2674890.7 (13.7) | <0.001 |
| 9-12 | 10949174.7 (29.8) | 1412016.8 (22.8) | 1047638.3 (35.1) | 2466845.1 (30.8) | 6022674.6 (30.8) |  |
| >12 | 15131944.0 (41.2) | 1125683.8 (18.2) | 1223208.7 (41.0) | 1955641.2 (24.4) | 10827410.3 (55.5) |  |
| **PIR** |  |  |  |  |  |  |
| Low <1.3 | 8429476.7 (23.0) | 3037689.7 (49.1) | 896744.4 (30.1) | 2417553.8 (30.1) | 2077488.9 (10.6) | <0.001 |
| Median(1.3-3.5) | 15571075.6 (42.4) | 2582098.7 (41.8) | 1494200.7 (50.1) | 3981262.9 (49.6) | 7513513.2 (38.5) |  |
| High (>=3.5) | 12711033.3 (34.6) | 564657.5 ( 9.1) | 589736.7 (19.8) | 1622665.6 (20.2) | 9933973.5 (50.9) |  |
| **Physical activity** |  |  |  |  |  |  |
| More | 18640948.5 (50.8) | 2174670.5 (35.2) | 1172275.7 (39.3) | 4069726.9 (50.7) | 11224275.4 (57.5) | <0.001 |
| Less | 4362056.5 (11.9) | 1334724.5 (21.6) | 918691.6 (30.8) | 831378.4 (10.4) | 1277262.0 ( 6.5) |  |
| Same | 13708580.6 (37.3) | 2675050.9 (43.3) | 889714.6 (29.8) | 3120376.9 (38.9) | 7023438.1 (36.0) |  |
| **Balance disorder** | 10735045.1 (29.2) | 3090130.1 (50.0) | 1232059.4 (41.3) | 2475747.8 (30.9) | 3937107.8 (20.2) | <0.001 |
| **Smokers** | 19618707.4 (53.4) | 2906068.5 (47.0) | 1563376.5 (52.5) | 4456467.0 (55.6) | 10692795.4 (54.8) | 0.108 |
| **Alcohol use** | 21960325.0 (59.8) | 2973185.9 (48.1) | 1328219.5 (44.6) | 4374096.3 (54.5) | 13284823.4 (68.0) | <0.001 |
| **CRP (mg/dL)** | 0.28 (0.13, 0.57) | 0.35 (0.17, 0.71) | 0.39 (0.19, 0.70) | 0.25 (0.11, 0.56) | 0.26 (0.12, 0.50) | 0.0001 |
| **History of cancer** | 7753540.3 (21.1) | 1363709.3 (22.1) | 640089.1 (21.5) | 1572818.8 (19.6) | 4176923.2 (21.4) | 0.839 |
| **History of CVD** | 8252392.9 (22.5) | 2180232.0 (35.3) | 747667.7 (25.1) | 2261146.4 (28.2) | 3063346.8 (15.7) | <0.001 |
| Heart disease | 7239391.0 (19.7) | 1872491.5 (30.3) | 595943.0 (20.0) | 1951834.9 (24.3) | 2819121.6 (14.4) | 0.0003 |
| Stroke | 1970345.2 ( 5.4) | 741882.7 (12.0) | 252693.4 ( 8.5) | 592118.1 ( 7.4) | 383651.0 ( 2.0) | <0.001 |
| **CMD risk factors** |  |  |  |  |  |  |
| 0 | 4342443.7 (11.8) | 506917.0 ( 8.2) | 184269.0 ( 6.2) | 909361.1 (11.3) | 2741896.5 (14.0) | 0.0003 |
| 1 | 12880201.4 (35.1) | 2176347.3 (35.2) | 907086.9 (30.4) | 2695154.9 (33.6) | 7101612.3 (36.4) |  |
| 2 | 12684451.0 (34.6) | 1886962.2 (30.5) | 972316.8 (32.6) | 3019795.5 (37.6) | 6805376.5 (34.9) |  |
| 3 | 5766429.1 (15.7) | 1322118.9 (21.4) | 701399.6 (23.5) | 1153492.0 (14.4) | 2589418.6 (13.3) |  |
| 4 | 1038060.4 ( 2.8) | 292100.5 ( 4.7) | 215609.5 ( 7.2) | 243678.7 ( 3.0) | 286671.6 ( 1.5) |  |
| Obestiy | 11611200.8 (31.6) | 1934132.5 (31.3) | 1318916.0 (44.2) | 2239931.5 (27.9) | 6118220.8 (31.3) | 0.005 |
| Hyperslipdemia | 16976070.6 (46.2) | 2762430.6 (44.7) | 1343190.0 (45.1) | 3713477.7 (46.3) | 9156972.3 (46.9) | 0.935 |
| Hypertension | 24911496.6 (67.9) | 4770158.5 (77.1) | 2341317.2 (78.5) | 5568762.9 (69.4) | 12231257.9 (62.6) | 0.001 |
| Diabetes | 6201864.2 (16.9) | 1618308.8 (26.2) | 814934.3 (27.3) | 1647764.6 (20.5) | 2120856.6 (10.9) | <0.0001 |
| **Gait speed (m/s)** | 1.0 (0.3) | 0.6 (0.1) | 0.7 (0.1) | 1.0 (0.1) | 1.1 (0.2) | <0.001 |
| **DSST (score)** | 46.9 (17.8) | 26.4 (10.5) | 51.9 (8.9) | 31.1 (8.8) | 59.1 (11.4) | <0.001 |
| **CMD mortality** | 8161837.7 (22.2) | 2172415.2 (35.1) | 1088063.6 (36.5) | 1963995.1 (24.5) | 2937363.8 (15.0) | <0.0001 |
| **All-cause mortality** | 23658945.2 (64.4) | 5356091.8 (86.6) | 2503961.3 (84.0) | 6045312.3 (75.4) | 9753579.8 (50.0) | <0.0001 |

**Abbreviations:** NHANES, National Health and Nutrition Examination Survey; PIR, poverty income ratio; CRP, C-reactive protein; CVD, cardiovascular and cerebrovascular disease; CMD, cardiometabolic disease; DSST, Digit Symbol Substitution Test, a cognitive test of processing speed;

The categorical variables are presented as number (percentage), mean (standard deviation) or median (interquartile range) for continuous variables.

**Supplementary Table 3** The weighted association of separated gait speed, processing speed and all-cause mortality

| **Models** | **All-cause Mortality** | | | |
| --- | --- | --- | --- | --- |
|  | **^a^Death no. /total no.** | | **HR (95%CI)** | ***P*-value** |
| **Gait speed** | Normal gait | Slow gait |  |  |
| Crude | 1023/1772 | 604/710 | 2.57 (2.17,3.05) | <0.001 |
| Model 1 | 1023/1772 | 604/710 | 1.79 (1.55, 2.06) | <0.001 |
| Model 2 | 1023/1772 | 604/710 | 1.60 (1.37, 1.87) | <0.001 |
| Model 3 | 1023/1772 | 604/710 | 1.54 (1.30, 1.81) | <0.001 |
| **Processing speed (DSST)** | Low DSST | High DSST |  |  |
| Crude | 701/1248 | 926/1234 | 2.22 (2.02,2.43) | <0.001 |
| Model 1 | 701/1248 | 926/1234 | 1.54 (1.37, 1.72) | <0.001 |
| Model 2 | 701/1248 | 926/1234 | 1.51 (1.35, 1.68) | <0.001 |
| Model 3 | 701/1248 | 926/1234 | 1.45 (1.31, 1.62) | <0.001 |

^a^Death and total number of participants are presented as unweighted number.

**Abbreviations:** DSST, Digit Symbol Substitution Test, a cognitive test of processing speed; HR, hazard ratio; CI, confidence interval

Model 1 was adjusted for age, sex, education, poverty income ratio (PIR);

Model 2 was additionally adjusted for physical activity, balance, smokers, alcohol use, C-reactive protein and history of cancer;

Model 3 was further adjusted for the history of cardiovascular and cerebrovascular diseases (CVD) and cardiometabolic disease risk factors (CMDRF)

**Supplementary Table 4** Competing risk model on cardiometabolic disease mortality

| **Variable** | **Death no. /total no.a** | **sHR (95% CI)** | | | |
| --- | --- | --- | --- | --- | --- |
|  |  | **Crude** | **Model 1** | **Model 2** | **Model 3** |
| **DSST (Per SD decline)** | 587/2482 | 1.33 (1.23, 1.44) | **1.2 (1.1, 1.31)** | 1.1 (0.98, 1.23) | 1.01 (0.9, 1.13) |
| **^a^Processing speed (DSST)** |  |  |  |  |  |
| High DSST (≥42) | 235/1248 | 1(Ref) | 1(Ref) | 1(Ref) | 1(Ref) |
| Low DSST (< 42) | 352/1234 | 1.64 (1.39, 1.93) | **1.33 (1.12, 1.57)** | 1.13 (0.93, 1.38) | 0.99 (0.81, 1.21) |
| **^b^Combined Gait-DSST** | |  |  |  |  |
| Group 1 | 164/1057 | 1(Ref) | 1(Ref) | 1(Ref) | 1(Ref) |
| Group 2 | 161/715 | 1.51 (1.22, 1.88) | **1.32 (1.06, 1.64)** | 1.18 (0.93, 1.5) | 1.05 (0.82, 1.34) |
| Group 3 | 71/191 | 2.83 (2.14, 3.74) | 2.42 (1.81, 3.24) | 2.28 (1.7, 3.06) | 1.91 (1.41, 2.59) |
| Group 4 | 191/519 | 2.86 (2.32, 3.53) | 2.1 (1.68, 2.63) | 1.82 (1.41, 2.34) | 1.47 (1.13, 1.92) |
| **^c^Processing speed (DSST)** |  |  |  |  |  |
| High DSST (>30) | 385/1821 | 1(Ref) | 1(Ref) | 1(Ref) | 1(Ref) |
| Low DSST (≤30) | 202/661 | 1.57 (1.32, 1.87) | **1.36 (1.13, 1.62)** | 1.15 (0.94, 1.42) | 1.06 (0.86, 1.3) |
| **^d^Combined Gait-DSST** | |  |  |  |  |
| Group 1 | 246/1445 | 1(Ref) | 1(Ref) | 1(Ref) | 1(Ref) |
| Group 2 | 79/327 | 1.49 (1.16, 1.91) | **1.4 (1.08, 1.8)** | 1.23 (0.93, 1.63) | 1.16 (0.88, 1.53) |
| Group 3 | 139/376 | 2.55 (2.07, 3.14) | 2.11 (1.69, 2.63) | 2 (1.6, 2.5) | 1.75 (1.39, 2.2) |
| Group 4 | 123/334 | 2.61 (2.09, 3.26) | 1.99 (1.57, 2.52) | 1.73 (1.34, 2.24) | 1.46 (1.12, 1.91) |

**Abbreviations:** DSST, Digit Symbol Substitution Test, a cognitive test of processing speed; sHR, subdistribution hazard ratio; CI, confidence interval.

^a^The definition of cognitive decline was below the value of median DSST (42).

^b^The combined group of gait-cognition was based on the cognitive decline (<42) and slow gait (<0.8m/s).

^c^The definition of cognitive decline was below the lowest quartile of DSST (30).

^d^The combined group of gait-cognition was based on the cognitive decline (<30) and slow gait (<0.8m/s).

Group 1: normal gait and high DSST; Group 2: normal gait and low DSST; Group 3: slow gait and high DSST; Group 4: slow gait and low DSST.

Model 1 was adjusted for age, sex;

Model 2 was additionally adjusted for education, poverty income ratio (PIR);

Model 3 was further adjusted for physical activity, balance, smokers, alcohol use, C-reactive protein (CRP), history of cancer, history of cardiovascular and cerebrovascular diseases (CVD), cardiometabolic disease risk factors (CMDRF).

**Supplementary Table 5** The weighted combined association of Gait-DSST on mortality (exclude participants who died within two years of follow-up)

| **Variable** | **^a^Death no. /total no.** | **HR (95% CI)** | | | |
| --- | --- | --- | --- | --- | --- |
|  |  | **Crude** | **Model 1** | **Model 2** | **Model 3** |
| **CMD Mortality** | **551/2389** |  |  |  |  |
| Group 1 | 158/1038 | 1(Ref) | 1(Ref) | 1(Ref) | 1(Ref) |
| Group 2 | 156/694 | 1.78 (1.38, 2.31) | 1.32 (0.98, 1.76) | 1.23 (0.92, 1.65) | 1.13 (0.85, 1.50) |
| Group 3 | 67/182 | 2.90 (2.05, 4.11) | 2.29 (1.62, 3.23) | 1.87 (1.28, 2.72) | 1.82 (1.26, 2.63) |
| Group 4 | 170/475 | 2.86 (2.34, 3.50) | 1.77 (1.41, 2.22) | 1.59 (1.25, 2.01) | 1.45 (1.14, 1.84) |
| **All-cause Mortality** | **1534/2389** |  |  |  |  |
| Group 1 | 523/1038 | 1(Ref) | 1(Ref) | 1(Ref) | 1(Ref) |
| Group 2 | 460/694 | 2.05 (1.81, 2.31) | 1.55(1.37, 1.74) | 1.57 (1.41, 1.75) | 1.50(1.34, 1.68) |
| Group 3 | 150/182 | 2.68 (2.10, 3.42) | 2.01 (1.57, 2.57) | 1.8 (1.39, 2.33) | 1.72 (1.32, 2.23) |
| Group 4 | 401/475 | 3.45 (2.81, 4.23) | 2.23 (1.85, 2.69) | 2.06 (1.69, 2.50) | 1.96 (1.61, 2.40) |

^a^Death and total number of participants are presented as unweighted number.

**Abbreviations: :** DSST, Digit Symbol Substitution Test, a cognitive test of processing speed; CMD, cardiometabolic disease; HR, hazard ratio; CI, confidence interval

Group 1: normal gait and high DSST; Group 2: normal gait and low DSST; Group 3: slow gait and high DSST; Group 4: slow gait and low DSST.

Model 1 was adjusted for age, sex, education, poverty income ratio;

Model 2 was additionally adjusted for physical activity, balance, smokers, alcohol use, C-reactive protein (CRP) and history of cancer;

Model 3 was further adjusted for the history of cardiovascular and cerebrovascular diseases (CVD) and cardiometabolic disease risk factors (CMDRF)

**Supplementary Table 6** The weighted combined association of Gait-DSST on mortality (exclude participants with missing covariate data)

| **Variable** | **^a^Death no. /total no.** | **HR (95% CI)** | | | |
| --- | --- | --- | --- | --- | --- |
|  |  | **Crude** | **Model 1** | **Model 2** | **Model 3** |
| **CMD Mortality** | **473/2006** |  |  |  |  |
| Group 1 | 138/884 | 1(Ref) | 1(Ref) | 1(Ref) | 1(Ref) |
| Group 2 | 142/595 | 1.85 (1.41, 2.43) | 1.40 (1.03, 1.90) | 1.3 (0.96, 1.77) | 1.19 (0.87, 1.62) |
| Group 3 | 58/150 | 2.93 (2.09, 4.10) | 2.35 (1.67, 3.30) | 1.85 (1.26, 2.72) | 1.77 (1.18, 2.65) |
| Group 4 | 135/377 | 2.65 (2.07, 3.39) | 1.71 (1.35, 2.15) | 1.47 (1.13, 1.92) | 1.31 (1.01, 1.74) |
| **All-Cause Mortality** | **1292/2006** |  |  |  |  |
| Group 1 | 445/884 | 1(Ref) | 1(Ref) | 1(Ref) | 1(Ref) |
| Group 2 | 404/595 | 2.15 (1.87, 2.47) | 1.64 (1.43, 1.89) | 1.65 (1.45, 1.89) | 1.58 (1.37, 1.81) |
| Group 3 | 123/150 | 2.53 (2.00, 3.19) | 1.86 (1.47, 2.37) | 1.65 (1.27, 2.14) | 1.52 (1.18, 1.97) |
| Group 4 | 320/377 | 3.47 (2.85, 4.24) | 2.28 (1.91, 2.72) | 2.05 (1.70, 2.49) | 1.96 (1.61, 2.38) |

^a^Death and total number of participants are presented as unweighted number.

**Abbreviations:** DSST, Digit Symbol Substitution Test, a cognitive test of processing speed; CMD, cardiometabolic disease; HR, hazard ratio; CI, confidence interval.

Group 1: normal gait and high DSST; Group 2: normal gait and low DSST; Group 3: slow gait and high DSST; Group 4: slow gait and low DSST.

Model 1 was adjusted for age, sex, education, poverty income ratio;

Model 2 was additionally adjusted for physical activity, balance, smokers, alcohol use, C-reactive protein (CRP) and history of cancer;

Model 3 was further adjusted for the history of cardiovascular and cerebrovascular diseases (CVD) and cardiometabolic disease risk factors (CMDRF)

**Supplementary Table 7** The weighted hazard ratios of CMD-specific and all-cause mortality by the combined Gait-DSST groups (incorporating Aspirin use and energy intake as confounding)

| **Variable** | **^a^**Death no. /total no. | **HR (95% CI)** | | |
| --- | --- | --- | --- | --- |
|  |  | **Crude** | **Model 1** | **Model 2** |
| **CMD Mortality** | **587/2482** |  |  |  |
| Combined Gait-DSST | | | | |
| Group 1 | 164/1057 | 1(Ref) | 1(Ref) | 1(Ref) |
| Group 2 | 161/715 | 1.74 (1.33, 2.27) | 1.09 (0.81, 1.47) | 1.1 (0.81, 1.73) |
| Group 3 | 71/191 | 2.92 (2.08, 4.10) | 1.86 (1.29, 2.68) | 1.85 (1.28, 2.69) |
| Group 4 | 191/519 | 2.88 (2.31, 3.58) | 1.40 (1.07, 1.83) | 1.38 (1.05, 1.81) |
| **All-Cause** Mortality | **1627/2482** |  |  |  |
| Combined Gait-DSST | | | | |
| Group 1 | 542/1057 | 1(Ref) | 1(Ref) | 1(Ref) |
| Group 2 | 481/715 | 2.06 (1.84, 2.31) | 1.51 (1.35, 1.68） | 1.50 (1.34, 1.68) |
| Group 3 | 159/191 | 2.70 (2.14, 3.40) | 1.72 (1.35, 2.18） | 1.72 (1.35, 2.19) |
| Group 4 | 445/519 | 3.54 (2.94, 4.26) | 2.01 (1.69, 2.39） | 1.97 (1.66, 2.35) |

^a^Death and total number of participants are presented as unweighted number.

**Abbreviations:** DSST, Digit Symbol Substitution Test, a cognitive test of processing speed; CMD, cardiometabolic disease; HR, hazard ratio; CI, confidence interval.

Group 1: normal gait and high DSST; Group 2: normal gait and low DSST; Group 3: slow gait and high DSST; Group 4: slow gait and low DSST.

Model 1 was adjusted for age, sex, education, poverty income ratio (PIR), physical activity, balance, smokers, alcohol use, C-reactive protein (CRP) and history of cancer, the history of cardiovascular and cerebrovascular diseases (CVD) and cardiometabolic disease risk factors (CMDRF) ;

Model 2 was further adjusted for Aspirin use and daily energy intake.

**Supplementary Figure 1** Stratified analysis of the association between the combined groups and all-cause mortality risk.

The stratification was adjusted for age, gender, education, family income, physical activity, balance, smokers, alcohol use, CRP, history of cancer, history of CVD, CMDRF except for the stratification factor itself. The circles represent the HRs and the horizontal lines represent 95% CIs. Diamonds represent the overall HR, and the outer points of the diamonds represent the 95% CI.

Group 1: normal gait and normal cognition; Group 2: normal gait and cognitive decline; Group 3: slow gait and normal cognition; Group 4: slow gait and cognitive decline.

**Abbreviations:** CRP, C-reactive protein; CVD, cardiovascular and cerebrovascular diseases; CMD, cardiometabolic disease; CMDRF, cardiometabolic risk factors; HR, hazard ratio; CI, confidence interval.
